# Bilateral Clavicle Fractures: A Case Report

**DOI:** 10.5704/MOJ.1803.015

**Published:** 2018-03

**Authors:** MY Bajuri, HW Boon

**Affiliations:** Department of Orthopaedics and Traumatology, Universiti Kebangsaan Malaysia, Cheras, Malaysia

**Keywords:** bilateral clavicle fracture, surgical intervention, excellent outcome

## Abstract

Bilateral clavicle fractures are not frequently seen. To treat these injuries surgically or non-surgically is still a debatable issue. Implant option for surgical management is also in doubt. We would like to share our experience in treating a patient with bilateral clavicle fracture surgically. He had excellent outcomes in terms of function and radiology. Surgical option for bilateral clavicle fractures promises excellent outcome in terms of early rehabilitation and return in function.

## Introduction

Clavicle fractures are common injuries. It has reported incidence of 2.4-4% in all type of fractures^1^. In young adults, it is encountered in 5-10% of injury^1^. However bilateral clavicle fracture is rare^2^. Bout *et al* searched all English-language journals published from year 1887-2010, and noted bilateral clavicle fractures comprised 0.43% of all clavicle fractures, with an overall incidence of between 0.011 to 0.017%^3^. As it is a rare injury, there is no general consensus to treat this injury surgically or non-surgically. If a patient is treated with open reduction and internal fixation, there is also a debatable issue of the type of implant best used in clavicle fracture. There are several options including anatomical locking plate, intramedullary K-wire, and non-locking 3.5mm reconstructive plate. Most of these patients have long term good functional outcome, whether treated surgically or non-surgically. We share our experience of a patient with bilateral clavicle fracture whom we treated with normal reconstructive 3.5mm plate.

## Case report

Mr. A, a 40-year old technician, allegedly involved in a motor vehicle accident, was admitted to our center. He was thrown from his motorcycle and was hit by a car from behind. There was no loss consciousness and no pain was noted elsewhere. There was no shortness of breath or chest pain. He was able to move all fingers, both wrists and elbows, and no other injuries were noted. On examination, both clavicles were tender. He was unable to raise both arms due to severe shoulder pain. Chest springing was negative.

His chest and shoulder radiographs showed bilateral clavicle fractures (Fig. 1a). He was offered surgical intervention in view of the nature of his job. He underwent bilateral clavicle plating simultaneously under general anaesthesia and propped up 30 degrees. Incisions were made directly on the clavicles and the fractures were reduced on direct vision. Non-locking 3.5mm reconstruction plates [Synthes, USA] were used. Reduction and fixations were checked with image intensifier. The wounds were closed with absorbable suture. Post-operation radiograph was also taken (Fig. 1b).

**Fig. 1a:**
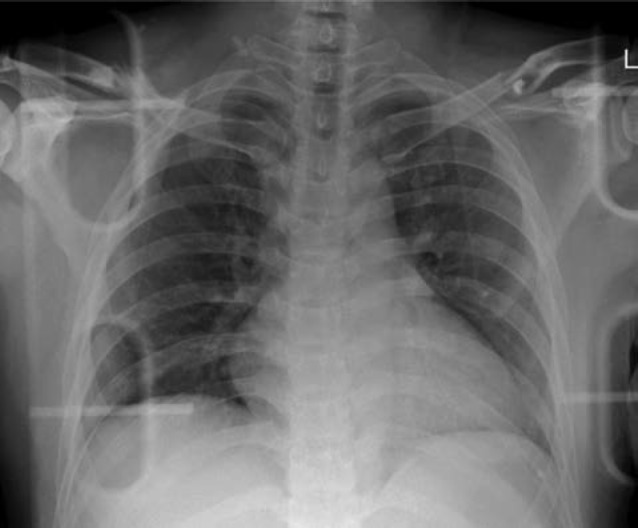
Bilateral clavicles fracture with no other injury seen. The radiograph was taken during patient was stabilized with arm board.

**Fig. 1b:**
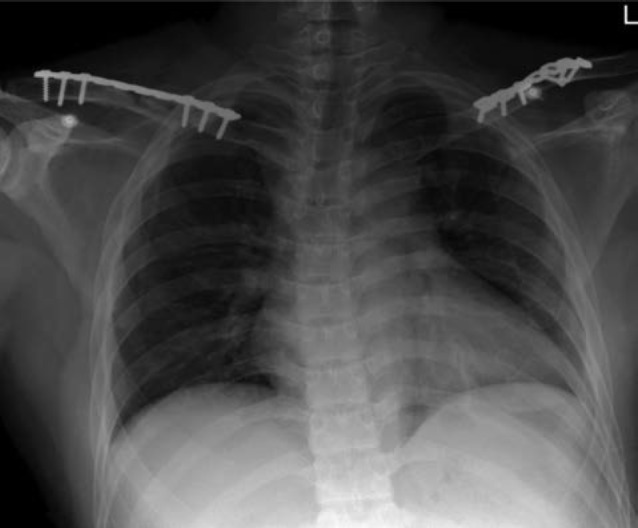
Radiographs immediate post-operation showed 3.5mm reconstruction plate used to fix the fractures.

Post-operation, patient was able to raise both his arms above his shoulders. Active ranges of motion were full with minimal pain. During follow-up one month post operatively, radiograph showed that the fractures had united and he was allowed to return to work on light duty. He returned to his normal daily activity two months’ post-operation. He was discharged from our follow-up at 5th month post-operation when he had achieved full union of the fractures, as shown in radiograph (Fig. 1c) and normal function.

**Fig. 1c:**
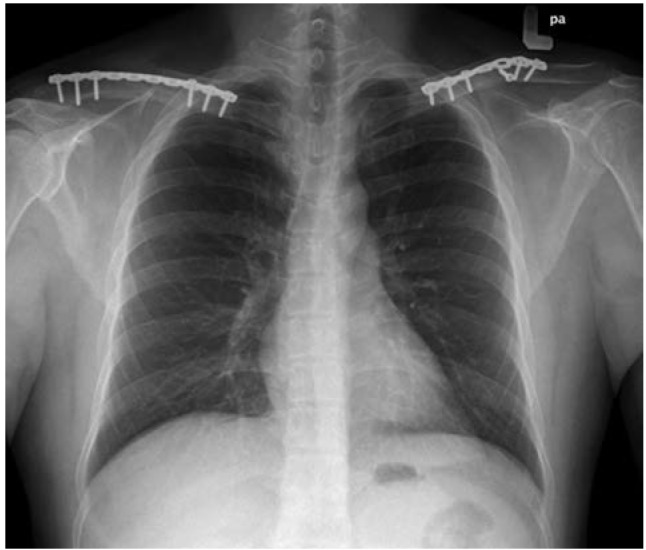
Union of fractures at 5th month post operation seen. As it is infrequently reported, the treatment of bilateral clavicle fractures is often not defined. Marya *et al* reported one of the largest series of bilateral clavicles fractures in the year 2002. Their cases (n=5) which were treated non-surgically, united in 6-8 weeks. Therefore, they suggested non-operative management for bilateral clavicle fractures^4^. However, these two series did not mention the duration that their patients underwent physiotherapy to achieve normal shoulder function and when they returned to work or normal daily activity.

## Discussion

Clavicle fracture is common but bilateral clavicle fractures are rare, contributing to as low as 0.43% of all clavicle fractures. However, this number can be underestimated, as clavicle fractures have always been paid least attention in poly-trauma cases. It is no surprise if bilateral clavicle fractures are altogether missed in such situations. In this patient, the bilateral clavicle fractures occurred almost at identical sites bilaterally at the lateral third of the clavicles.

On general orthopaedic principle, all fractures should be immobilized, as fracture movement will cause delayed union or non-union. Furthermore, any mobile fracture in acute phase will cause pain and discomfort. When bilateral clavicle fractures are managed without operation, the patient will experience more pain and become more incapacitated. These will cause more stiffness in short term. Therefore, we decided to proceed with surgical intervention for our patient.

Implant option is another debatable issue. Anatomical locking plate is a good option for treating clavicle fractures. van Den Bout suggested anatomical locking plate in treating bilateral clavicles fractures, especially in the elderly group or osteoporotic bone^3^. In another study Bajuri *et al* concluded that there was a need for surgical intervention to treat clavicle fractures as it would improve shoulder functional outcomes^5^. In our case, we decided to use 3.5mm reconstruction plate to fix the fractures as this implant could be contoured to achieve bone alignment. Furthermore, our patient had good bone quality and did not need extra screw to avoid pull-out force. The patient was able to raise both upper limbs above shoulder level on Day 1 post-operation. His fractures achieved union in one-month after fixation with no limitation in range of shoulder motion. He was able to return to work at that point of time. The repeat radiograph at 5th month post-operation showed fully united clavicle fractures with no evidence of implant pull out.

Therefore, non-anatomical non-locking 3.5mm reconstruction plate is a good option as it is a more affordable implant in our setting. Since both surgical or non-surgical management have advantages and disadvantages, patient must be counselled in order to achieve shared decision making.
